# Physician Assessment and Feedback During Quality Circle to Reduce Low-Value Services in Outpatients: a Pre-Post Quality Improvement Study

**DOI:** 10.1007/s11606-021-06624-9

**Published:** 2021-02-08

**Authors:** Omar Kherad, Kevin Selby, Myriam Martel, Henrique da Costa, Yann Vettard, Philippe Schaller, Marc-André Raetzo

**Affiliations:** 1 Internal Medicine Department, Hôpital de la Tour and University of Geneva, 1217 Geneva, Switzerland; 2 Center for Primary Care and Public Health (Unisanté), Lausanne, Switzerland; 3 Division of Epidemiology, McGill University Health Centre, McGill University, Montréal, Québec, Canada; 4 Réseau Delta, HMO, Geneva, Switzerland

**Keywords:** Choosing Wisely, low-value care, educative feedback, overuse

## Abstract

**Background:**

The impact of the Choosing Wisely (CW) campaign is debated as recommendations alone may not modify physician behavior.

**Objective:**

The aim of this study was to assess whether behavioral interventions with physician assessment and feedback during quality circles (QCs) could reduce low-value services.

**Design and Participants:**

Pre-post quality improvement intervention with a parallel comparison group involving outpatients followed in a Swiss-managed care network, including 700 general physicians (GPs) and 150,000 adult patients.

**Interventions:**

Interventions included performance feedback about low-value activities and comparison with peers during QCs. We assessed individual physician behavior and healthcare use from laboratory and insurance claims files between August 1, 2016, and October 31, 2018.

**Main Measures:**

Main outcomes were the change in prescription of three low-value services 6 months before and 6 months after each intervention: measurement of prostate-specific antigen (PSA) and prescription rates of proton pump inhibitors (PPIs) and statins.

**Key Results:**

Among primary care practices, a QC intervention with physician feedback and peer comparison resulted in lower rates of PPI prescription (pre-post mean prescriptions per GP 25.5 ± 23.7 vs 22.9 ± 21.4, *p* value<0.01; coefficient of variation (Cov) 93.0% vs 91.0%, *p*=0.49), PSA measurement (6.5 ± 8.7 vs 5.3 ± 6.9 tests per GP, *p*<0.01; Cov 133.5% vs 130.7%, *p*=0.84), as well as statins (6.1 ± 6.8 vs 5.6 ± 5.4 prescriptions per GP, *p*<0.01; Cov 111.5% vs 96.4%, *p*=0.21). Changes in prescription of low-value services among GPs who did not attend QCs were not statistically significant over this time period.

**Conclusion:**

Our results demonstrate a modest but statistically significant effect of QCs with educative feedback in reducing low-value services in outpatients with low impact on coefficient of variation. Limiting overuse in medicine is very challenging and dedicated discussion and real-time review of actionable data may help.

**Supplementary Information:**

The online version contains supplementary material available at 10.1007/s11606-021-06624-9.

## INTRODUCTION

Quality circles (QCs) are small groups of general physicians (GPs), typically one moderator and five to ten participants from similar backgrounds, who meet at regular intervals to discuss and review their clinical practice and solve quality-oriented medical problems. QCs are an ideal platform to give individual performance feedback and peer comparisons in a trusting environment. Interactions between peers promote collaborations, partnership, and education to optimize the quality of care and disseminate best practice guidelines, such as those published by the Choosing Wisely (CW) campaign.^1,2^. Since the launch of this campaign, several medical and surgical specialty societies have indeed produced top-five lists with hundreds of recommendations to stimulate conversations between physicians and patients about unnecessary tests, treatments, and procedures that account for up to 20–30% of all medical costs.^2,3^ These top-five lists draw attention to low-value services, but they must be translated into measurable recommendations and valid quality indicators if we hope to assess their effect on physician behavior. Indeed, the dissemination of guidelines alone does not appear to change physician behavior.^4–6^

When comparing different approaches to nationwide implementation of CW recommendations, data measurement is essential in order to assess whether physicians really follow the recommendations in their routine clinical practice.^7,8^ Recent studies have demonstrated the feasibility of directly measuring low-value services that provide minimal benefits for patients.^8,9^ An accepted alternative means of assessing physicians’ use of low-value care is to identify variations in medical practice between physicians.^10^ Variations in the use of medical care can reveal significant differences between regions or physicians that are warning signs of overuse and strong quality indicators.^9,11,12^ Once variation is identified, some behavioral interventions with data feedback and peer comparison can result in lower use of low-value care such as inappropriate antibiotic prescribing for acute respiratory tract infections among primary care practices.^13^

The present study examined the use of healthcare services related to recommendations appearing on top-five lists released in the Swiss CW campaign^3^. The aim was to assess the variation at which use of these low-value services occurred, and to determine whether behavioral interventions (physician assessment and feedback) during QCs as a support tool may change the prescription of these low-value services among primary care practices.

## METHODS

### Design of the Study

We performed a pre-post intervention study with a parallel comparison group to compare changes in the use of low-value services before and after the intervention and thus measure the impact of the intervention during QCs. The baseline period included prescriptions that occurred 6 months before the intervention and the post-intervention period also analyzed over a 6-month period. An additional month-by-month analysis was performed to evaluate the sustainability of the intervention over time.

Institutional review board approval was granted by the Commission Cantonale d’Ethique de la Recherche (CCER) on the Use of Human Subjects.

### Study Population

#### Setting and Inclusion Criteria

We assessed physician behavior and healthcare use from Delta Network enrollment and claims files from August 1, 2016, through October 31, 2018, fee-for-service beneficiaries.

The Delta Network is a health maintenance organization (HMO) established in the west part of Switzerland with 4 areas that limit member coverage to medical care provided through a network of 700 GPs who are under contract with the HMO. The Delta Network takes care of more than 250,000 insured adults (>18 years old) and persons contracting with all Swiss health insurance companies. Delta Network physicians agreed to be accountable for the quality, cost, and overall care of HMO beneficiaries who are enrolled in the traditional fee-for-service program who are assigned to it. All patients who contracted with Delta Network were included, subject to data access as the majority, but not all, laboratories and insurance companies’ partners agreed to transmit the data, making the information only accessible to approximately 150,000 of 250,000 insured patients.

### Data Analysis

#### Study Variables

We focused on treatments and procedures that are frequently overused, feasible to change, and in the domain of the Swiss CW campaign^3^. Each item could be analyzed via insurance and laboratory claims data.

- Prescribing rate of proton pump inhibitors (PPIs), reflecting the CW recommendation to not continue long-term treatment with proton pump inhibitors without titrating to the lowest effective dose needed. (A list of PPIs is available in Appendix 1.)
- Prescribing rate of prostate-specific antigen (PSA) in men and in subcategory of men >75 years old, reflecting the CW recommendation to not screen for prostate cancer without a shared decision discussion and not to screen over age 75. These data were provided by our partner laboratory UNILABS which deals with more than 50% of the requests of the physicians in the network.
- Prescribing rate of statins in patients >75 years old reflecting the CW recommendation to avoid statins for primary prevention of nondiabetic patients over age 75. (A list of statins is available in Appendix 1.)

#### Behavioral Intervention

Following the implementation of the Swiss CW campaign ^3^, thematic QCs were instituted to target the overuse of low-value services in ambulatory practices. We considered interventions that have been characterized as low-value by the “Swiss CW campaign”.^3^ These interventions have been found to provide little to no clinical benefit on average, either in general or in specific clinical scenarios. The organization of thematic QCs is described in Table 1. These thematic QCs took place systematically over a period of 2–3 months: (i) from February 1 to April 30 2018 for PPIs, (ii) from February 1 to March 30 2017 for statins, and (iii) from February 1 to April 30 2017 for PSA. As a first experience, the QC on PSA required an extended period to reach all physicians from the network. An additional thematic QC on PSA was done later in 2017; therefore, the intervention period was extended from February 1, 2017, to February 28, 2018. During these intervention periods, each physician attended at least one thematic QC (1-h session) that addresses each topic separately.

**Table 1:** Thematic Quality Circle (QC) Process Step-by-Step

1. Identification of a situation of low-value service 2. Creation of clinical case scenario 3. Presentation of the clinical case during the QC by the moderator 4. Participants are invited to give their opinion and behavior in the situation presented, without intervention of the moderator 5. The moderator uses variation in care between participants to start the discussion and presents the latest recommendations for good clinical practice 6. Data reporting on variation of low-value services within the network and benchmarking at participants and the QC level 7. Discussion of intervention measures to avoid use of the low-value service within the group (nudge, clinical decision tool)	

#### Outcome Measures

The primary outcome was to assess the mean prescription change of these low-value services within the network after the behavior interventions. Secondary outcome was to assess the coefficient of variation (Cov) to measure the dispersion of data across the mean per GP. The comparison group was a network of physicians who did not attend to any QC during the study period.

#### Data Sources

The data are transmitted to the network by the insurers within the framework of the health insurance contract with the Delta Network. Data exchanges between the insurance company and the network are fully secured (e-mail HIN, SFTP with RSA certificate, HTTPS website with dual authentication). The data used in the current study are an anonymized version of the initial data.

### Statistical Analysis

Descriptive usage analyses were performed (mean, standard deviation, and median) to describe the study population and drug and laboratory prescription rates. Data were compared using *t* tests for continuous variables and chi-square tests for categorical variables for matched group, each GP being his own control before and after the intervention. In order to limit secular trend, we compared the prescribing behavior of GPs who did not attend to QC (zero QC) during the same period with those who benefited from thematic QCs. The coefficient of variation (Cov) was calculated to indicate the degree of variation (ratio of the standard deviation to the mean, expressed as a percentage (%)): a low Cov (usually <15%) indicates relatively little variation within the sample and a higher Cov indicates more variation. The test by Forkman was used to calculate *p* values of Cov with significance level at 0.05. No attempt at imputation of missing data was carried out. All analyses were performed using SAS version 9.4 (Cary, NC, USA).

## RESULTS

Demographics data of GPs who attended QCs are depicted in Table 2. Data were similar to those who did not attend QCs on each intervention, except a higher percentage of woman physicians in the former group (Appendix 2).

**Table 2:** Demographics Data of General Physicians (GPs) Who Attended Quality Circles

	GPs in the PPI group, *N*=748	GPs in the PSA group, *N*=150	GPs in the statin group, *N*=639
Age (years, mean SD)	51.3 ± 9.8	59.9 ± 5.5	52.7 ± 9.3
Women	314 (24.0%)	50 (33.3%)	258 (40.4%)
Network area			
1	10 (1.3%)	0 (0.0%)	1 (0.2%)
2	446 (59.7%)	127 (84.7%)	392 (61.3%)
3	18 (2.4%)	0 (0.0%)	10 (1.6%)
4	273 (36.6%)	23 (15.3%)	235 (36.8%)
Years since graduation	25.8 ± 9.9	32.6 ± 7.1	26.5 ± 10.0
≤ 5 years	1 (0.78)	0 (0.0%)	1 (0.8%)
> 5 years ≤ 10 years	6 (4.7%)	0 (0.0%)	5 (3.9%)
> 10 years	121 (95.5%)	35 (100.0%)	122 (95.3%)

### PPIs

**Figure 1:**
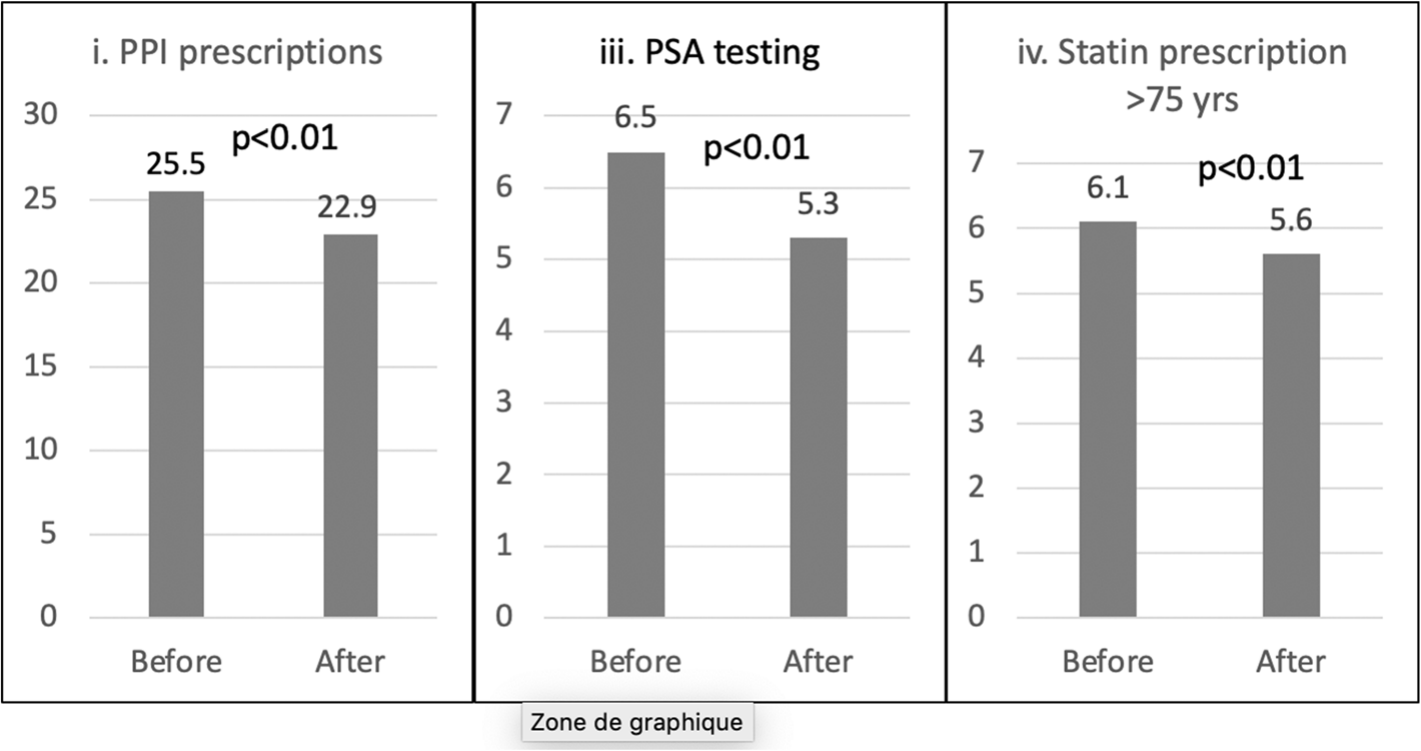
Change in the average number of prescriptions per GP of three low-value measures per GP before and after behavioral interventions.

A total of 28,750 PPI prescriptions were filled by 483 GPs participating in a QC during the study period. Thematic QCs on PPI prescription were conducted from February 1, 2018, to April 30, 2018 (3 months of behavior intervention). In the pre-intervention period, an average of 25.5 ± 23.7 (median: 19.0) prescriptions per GP were filled compared to 22.9 ± 21.4 (median: 18.0) in the post-intervention period (*p*≤0.01) (Fig. 1). In total, 52.0% of physicians decreased the number of prescriptions following the intervention period. The coefficient of variation was not significantly different between the pre- and post-intervention (93.0% vs 91.9%, *p*=0.49).

In comparison, the group of physicians (*n*=265) who did not attend the behavior intervention QC prescribed 11,700 PPIs during the study period without a significant change over time (18.2 ± 25.0 (median: 19.0) pre-intervention prescriptions per GP vs 17.4 ± 36.0 (median: 18.0) post-intervention prescriptions, *p*=0.54).

### PSA prescription

During the study period, 3484 PSA tests were prescribed by 118 GPs. The thematic QC with data feedback on PSA prescription took place from February 1 to April 30 2017 (3 months of behavior interventions) but since there were additional thematic QCs later in 2017, the intervention period was extended from February 1, 2017, to February 28, 2018. In the pre-intervention period, the mean PSA prescription per GP was 6.5 ± 8.7 (median 3.0) as significantly higher compared to 5.3 ± 6.9 (median 3.0) in the post-intervention period (*p*=0.03) (Fig. 1). In total, 56.0% of physicians decreased the number of prescriptions following the intervention period. The coefficient of variation was 133.5% for the pre-intervention period and 130.7% for the post-intervention period (*p*=0.84).

When restricting this analysis to patients >75 years old (total of 273 PSA prescriptions by 61 physicians), results were similar (2.7 ± 2.7 prescriptions per GP in the pre-intervention period compared to 1.8 ± 2.1 in the post-intervention period (mean difference: *p*=0.04). The coefficient of variation was 101.2% in the pre-intervention period to 114.2% in the post-intervention period (*p*=0.50).

In comparison, the group of physicians (*n*=32) who did not attend the behavior intervention QCs did 621 PSA tests during the study period without any significant change over time (2.9 ± 3.6 per GP vs 3.8 ± 3.5 per GP, *p*=0.19).

### Statins

A total of 15,692 statin prescriptions were filled by 384 GPs participating in a QC during the study period. Thematic QCs on statin prescription were conducted from February 1, 2018, to March 31, 2017 (2 months of behavior intervention).

In the pre-intervention period, for patients >75 years old, an average of 6.1 ± 6.8 (median: 4.0) prescriptions per GP were filled compared to 5.6 ± 5.4 (median: 4.0) in the post-intervention period (*p*<0.01) (Fig. 1). There was not a significant change among physicians who did not attend a QC (5.6 ± 6.8 per GP pre-intervention vs 5.4 ± 6.7 per GP post-intervention (*p*=0.29).

In total, 47.5% of physician decreased the number of prescriptions following the intervention period. The coefficient of variation was 111.5% in the pre-intervention period and 96.4% in the post intervention period (*p*=0.21).

## DISCUSSION

This study is the first to evaluate the impact of QCs as an additional tool to implement recommendations from the CW campaign using region-wide population-level data from insurance and laboratory claims in Switzerland. Our results suggest that among primary care practices, thematic QC intervention with data feedback and peer comparison resulted in lower mean of PPI prescription (25.5 ± 23.7 per GP vs 22.9 ± 21.4 per GP, *p*<0.01), PSA (6.5 ± 8.7 per GP vs 5.3 ± 6.9 per GP, *p*=0.03), and statin prescriptions over age 75 years (6.1.0 ± 6.8 per GP vs 5.6 ± 5.4 per GP, *p*<0.01). Decreases in prescriptions were similar when restricting the analysis for 75 years old for PSA. The majority of physicians decreased their number of prescriptions following the intervention period (52% for IPP, 56.0% for PSA, and 64.0% for statin). Conversely, we did not observe significant decreases in the comparison group who did not attend QCs suggesting that observed differences cannot be explained by secular trends in decreasing number of prescriptions. The coefficient of variation was very high among study groups indicating a large dispersion of prescription rate. This coefficient decreased after the intervention but without reaching statistical significance.

Measuring the impact of CW efforts to eliminate waste in healthcare is complex and requires a variety of approaches. Simply informing doctors to order tests parsimoniously has limited effect given administrative barriers to change and accurate documentation remain suboptimal. ^7,14^ Creating the CW top-five lists raised awareness among different stakeholders but it was only a first step: such lists must be translated into measurable recommendations to assess their effect on changing behavior. ^6,15,16^ Subsequently, CW published a framework based on physician attitudes, behaviors, and patient engagement to measure its effectiveness going forward.^17,18^ Without robust and reliable data reporting, most physicians cannot appreciate the extent to which they are contributing to overuse. Accessing claims data in our study allowed an evaluation of CW recommendations on low-value services based on region-wide population-level data and on a practical level. Only few data have assessed effectively the impact of CW campaign in ambulatory setting. A US analysis of database compensation claims published in 2015 examined the impact of seven CW recommendations with contrasting results: there were clinically significant reductions in two tests (imaging for headache, cardiac imaging), a small increase in two recommendations (nonsteroidal anti-inflammatory drug use and screening of very young women for the human papillomavirus), and statistically significant but clinically insignificant decreases in the remaining measures.^6^

Dissemination of guidelines by itself is insufficient to drive practice change, which requires more robust implementation strategies in regard to the complexities of different practice environments. There is an increasing interest in the use of behavioral science to affect practice in medicine. Reporting performance data back to physicians with educative feedback can be used as a “radar sensor effect” similar to traffic controlling, in order to nudge physicians’ behaviors.^15,19^ Our pragmatic quality improvement study introduced the use of a behavioral intervention with data feedback during QCs to reduce low-value services in the outpatient setting.

QCs are an ideal platform and context to provide personal performance feedback and peer comparisons. Even if reported effectiveness for improving quality in healthcare varies substantially among studies assessing their impact, QCs are a vehicle for discussing issues and reflecting on practice. ^1,20^ QCs may also improve individual and group performance by reducing costs, encouraging professionals to order fewer but more appropriate tests, improving prescription habits, and reporting critical incidents^20^. During QCs, dedicated discussion and review time of actionable data may help to disseminate best practice guidelines, such as the top-five lists published by the CW campaign. A study confirmed that data reporting with educative feedback has a positive effect and reduced the level of inappropriate care.^13^ In that study, the use of accountable justification and peer comparison as behavioral interventions resulted in lower rates of inappropriate antibiotic prescribing for acute respiratory tract infections among primary care practices.^13^ Audit and feedback combined with multifaceted outreach education for healthcare professionals have also been successful in primary care in Australia.^21^

One of the challenges in measuring progress in reducing overuse is to identify when a service was provided inappropriately because the definition of appropriateness of a service often includes knowing about symptoms and physical exam findings often not included in administrative databases. Furthermore, many of the CW recommendations are clinically nuanced, and data systems lack the precision to measure accurately. To address the issue of appropriateness, we also assessed the Cov of these low-value services as a secondary outcome that reveals to be lower after our intervention but did not reach statistical significance. Clinical variation is an accepted way to identify potential overuse in medicine among practitioners.^11,22^ Variation analyses can show significant differences that are warning signs of overuse and a strong quality indicator. Clinical variation can act as catalysts for change by stimulating debate, engaging all participants in the health system so that patient-focused care can be achieved.^11^ Process standardization can dramatically decrease variation and eventually improve performance.^22,23^ Furthermore, the case mix of studied populations has little importance when interpreting variation results, which avoids some bias and makes benchmarking between physician easier. Our low impact on coefficient of variation reflects the challenge of changing clinician decision-making, particularly in the ambulatory setting. As we do not have yet access to diagnoses and clinical context through electronic health records, we gave feedback to physicians about their performance based on utilization data, using the variation of practice among physicians as estimates of appropriate adherence.

### Limitations of the Study

Our study has several limitations. Our analysis is based on administrative claims data that do not adequately capture the clinical circumstances that led to ordering a service, which may be essential for some recommendations, such as implying a shared decision-making process like PSA test measurement. Therefore, we cannot affirm these interventions were inappropriate for all individual patients. To assess the variability of our findings across a spectrum of these important measurement properties, we stratified the measures of PSA dosage by age (>75 years old), for detecting low-value care. Limiting the prostate cancer screening measure to beneficiaries over age 75 instead of 50 increases its specificity of being inappropriate (smaller proportion of appropriate services misclassified as inappropriate).

The relative decrease of variation may reflect a standardization of medical use but does not permit to distinguish inappropriate from appropriate use. Besides, some variation in healthcare delivery is warranted and desirable, such as meeting differences in patients’ health needs or health preferences.

Notwithstanding the issue of appropriateness and measurement, our intervention studies focus on a brief period after the change, limiting the capacity of physician to modify their practice and deprescribe some drugs such as statin. Furthermore, even if we did comparison with a group of physicians who did not attend the behavioral intervention, it is possible that other factors may have been responsible for changes of low-value services such as different case mix or the numbers of visits per GP. Self-selection of physicians likely introduced a selection bias, as clinicians participating in QCs are probably more amenable to changing their practice patterns than our comparison group that did not participate in QCs. Furthermore, a Hawthorne effect—the tendency for some to perform better when they perceive that their work is under scrutiny—may at least partly explain the observed positive effect of the thematic QC in our study. Eventually, our comparison group was quite small, limiting statistical precision for before-after comparisons.

## CONCLUSION

The central goal of the CW campaign was to change the culture of medical care that has historically supported overuse of unnecessary tests, treatments, and procedures. This study using an educative data feedback during QCs as a support tool provides a starting point for further evaluation of the influence of the initiative on changing behavior by analyzing changes in volume and variation of these low-value services. Educative feedback can provide the opportunity for physicians to carry out analyses of their own practices and to see whether there are opportunities for implementing higher value practices. The relatively modest change suggests though that additional long-term interventions are necessary for wider implementation of CW campaign.

## Supplementary Information

ESM 1 (DOCX 25 kb)
