# Supplementary material for: Physician Assessment and Feedback During Quality Circle to Reduce Low-Value Services in Outpatients: a Pre-Post Quality Improvement Study
Source: J Gen Intern Med. 2021 Feb 8;36(9):2672–7. doi: 10.1007/s11606-021-06624-9 (PMC8390713; doi:10.1007/s11606-021-06624-9)
Supplement: Supplementary file 1 — (DOCX 25 kb) [file 11606_2021_6624_MOESM1_ESM.docx]

Appendix1:

PPI

| **1** | **2** | **3** | **4** | **5** |  |
| --- | --- | --- | --- | --- | --- |
|  |  |  |  |  |  |
| **esoméprazole** | **lansoprazole** | **oméprazole** | **pantoprazole** | **rabéprazole** | **dexlansoprazole** |
| ESOMEPRAZOLE | LANSOPRAZOLE | OMEPRAZOLE | PANTOPRAZOLE | RABEPRAZOLE | DEXILANT |
| ESOMEPRAZOL | LANSOPRAZOL | OMEPRAZOL | PANTOPRAZOL | PARIET |  |
| ESOMEP | AGOPTON | ANTRAMUPS | ACIDO-X |  |  |
| ESOPRAX | LANSOPRAX | OMED | PANTOFELAN |  |  |
| NEXIUM |  | OMEPRAX | PANTOZOL |  |  |
| VIMOVO |  |  | PANPRAX |  |  |

Statins:

|  |  |  |  |  |  |
| --- | --- | --- | --- | --- | --- |
| **1** | **2** | **3** | **4** | **5** |  |
|  |  |  |  |  |  |
| **simvastatine** | **pravastatine** | **fluvastatine** | **atorvastatine** | **rosuvastatine** | **pitavastatine** |
| SIMVASTATINE | PRAVASTATINE | FLUVASTATINE | ATORVASTATINE | ROSUVASTATINE | LIVAZO |
| SIMCORA | MEVALOTIN | FLUVASTATIN | ATORVASTATIN | CRESTASTATIN |  |
| SIMVASINE | SELIPRAN | LESCOL | ATORVA | CRESTOR |  |
| ZOCOR | PRAVASTAX |  | ATORVASTAX | ROSUVASTATIN |  |
| CHOLIB |  |  | SORTIS | ROSUVASTAX |  |
| INEGY |  |  | CADUET | ROSUVAST |  |
|  |  |  | ATOZET |  |  |
|  |  |  | TRIVERAM |  |  |

Appendix 2

**Demographic data of general physician (GP) who attended thematic quality circle (QC) compared to those who did not**

1. **Proton pumps inhibitors (PPI)**

|  | **All GP**  **N=748** | **GP who attended QC**  **N=483** | **GP who did not attend QC**  **N=265** | p-value |
| --- | --- | --- | --- | --- |
| **Age (years), mean SD*** | 51.3 ± 9.8 | 51.9 ± 9.7 | 50.0 ± 9.8 | 0.10 |
| **Women** | 314 (24.0%) | 241 (50.0%) | 73 (27.5%) | **<0.01** |
| **Network** |  |  |  |  |
| **1** | 10 (1.3%) | 3 (0.6%) | 7 (2.6%) | 0.04 |
| **2** | 446 (59.7%) | 292 (60.6%) | 154 (58.1%) | 0.53 |
| **3** | 18 (2.4%) | 10 (2.1%) | 8 (3.0%) | 0.42 |
| **4** | 273 (36.6%) | 177 (36.7%) | 96 (36.2%) | 0.91 |
| **Years since graduation** | 25.8 ± 9.9 | 24.6 ± 9.9 | 28.6 ± 9.5 | 0.34 |
| **≤ 5 years** | 1 (0.78) | 1 (1.2%) | 0 (0.0%) | 1.00 |
| **> 5 years ≤ 10 years** | 6 (4.7%) | 5 (5.6%) | 1 (2.6%) | 0.67 |
| **> 10 years** | 121 (95.5%) | 83 (93.3%) | 38 (97.4%) | 0.67 |

*412 missing values

1. **Prostate specific antigen (PSA)**

|  | **All GP**  **N=150** | **GP who attended QC**  **N=118** | **GP who did not attend QC**  **N=32** | p-value |
| --- | --- | --- | --- | --- |
| **Age (years)*, mean SD** | 59.9 ± 5.5 | 59.6 ± 6.6 | 63.4 ± 5.7 | 0.25 |
| **Women** | 50 (33.3%) | 46 (39.0%) | 4 (12.5%) | **<0.01** |
| **Network** |  |  |  |  |
| **1** | 0 (0.0%) | 0 (0.0%) | 0 (0.0%) | 1.00 |
| **2** | 127 (84.7%) | 101 (85.6%) | 26 (81.3%) | 0.58 |
| **3** | 0 (0.0%) | 0 (0.0%) | 0 (0.0%) | 1.00 |
| **4** | 23 (15.3%) | 17 (14.4%) | 6 (18.8%) | 0.58 |
| **Years since graduation** | 32.6 ± 7.1 | 30.7 ± 6.4 | 37.4 ± 6.6 | <0.01 |
| **≤ 5 years** | 0 (0.0%) | 0 (0.0%) | 0 (0.0%) | 1.00 |
| **> 5 years ≤ 10 years** | 0 (0.0%) | 0 (0.0%) | 0 (0.0%) | 1.00 |
| **> 10 years** | 35 (100.0%) | 25 (100.0%) | 10 (100.0%) | 1.00 |

*87 missing values

1. **Statin**

|  | **All GP**  **N=639** | **GP who attended QC**  **N=383** | **GP who did not attend QC**  **N=265** | p-value |
| --- | --- | --- | --- | --- |
| **Age (years)*, mean SD** | 52.7 ± 9.3 | 52.6 ± 9.6 | 52.9 ± 8.6 | 0.82 |
| **Women** | 258 (40.4%) | 188 (49.1%) | 70 (27.5%) | **<0.01** |
| **Network** |  |  |  |  |
| **1** | 1 (0.2%) | 0 (0.0%) | 1 (0.4%) | 0.20 |
| **2** | 392 (61.3%) | 240 (62.5%) | 152 (59.6%) | 0.46 |
| **3** | 10 (1.6%) | 8 (2.1%) | 2 (0.8%) | 0.33 |
| **4** | 235 (36.8%) | 135 (35.2%) | 100 (39.2%) | 0.29 |
| **Years since graduation** | 26.5 ± 10.0 | 25.7 ± 9.9 | 27.7 ± 10.2 | 0.26 |
| **≤ 5 years** | 1 (0.8%) | 1 (1.3%) | 0 (0.0%) | 1.00 |
| **> 5 years ≤ 10 years** | 5 (3.9%) | 2 (3.9%) | 2 (3.9%) | 1.00 |
| **> 10 years** | 122 (95.3%) | 73 (94.8%) | 49 (96.1%) | 1.00 |

*351 missing values
